# Paradoxical changes in lumen size during progression and regression of carotid atherosclerosis

**DOI:** 10.1186/1532-429X-14-S1-O43

**Published:** 2012-02-01

**Authors:** Sobhan Kodali, Mark Doyle, Saundra Grant, David Neff, Ronald B Williams, June Yamrozik, Geetha Rayarao, George Angheloiu, Vikas K Rathi, Robert W Biederman

**Affiliations:** 1 Allegheny General Hospital, Pittsburgh

## Summary

Atherosclerotic changes regarding the lipid pool and frbrous cap are related to the lumen and show countgerintuitive changes; a discourse on Glagov's Hypothesis.

## Background

Relative changes in arterial lumen size during progression and regression of atherosclerosis particularly in response to lipid modulating therapy are largely unknown. This study was designed to investigate the effects of statin therapy on human carotid atherosclerotic lesions as measured by high-resolution Cardiac Magnetic Resonance (CMR).

## Methods

Via CMR (1.5T GE, WI), 35 carotid arteries of 18 asymptomatic, ‘statin naïve’ patients with maximum carotid stenosis >50% (mean 64±21), were imaged at baseline and following 12 months of randomized, blinded statin therapy (simvastatin 40mg or Vytorin (Ezetimibe 10mg/ simvastatin 40mg)). The effects of statins on these lesions were evaluated as changes in carotid outer wall area (OWA), lumen area (LuA), vessel wall area (VWA), lipid area (LpA) and lipid percentage (Lp%). The percentage of stenosis of each slice was determined in reference to a normal or near normal sl.

## Results

A total of 707-2mm slices of interpretable quality were available representing 39 in vivo plaques. Among these as followed over time, 378 (53.5%) demonstrated progression while 329 (46.5%) showed regression. Among ‘Progressors’, OWA, VWA, Lp% and LuA increased (P< .001 for OWA, VWA and Lp% and 0.14 for LuA), while among ‘Regressors’, all the above parameters decreased significantly (P<.001 for all). There was no statistically significant change in any of the parameters when all contiguous slices of all carotid plaques were analyzed in a 3D fashion.

## Conclusions

Changes in carotid plaque after lipid lowering therapy, as measured by high-resolution CMR depict a counterintuitive effect on lumen size: as VWA and LpA decrease so does the LuA. Similarly, as VWA and LpA increase so does LuA. The measurement of luminal area may not be a reliable measure of response of carotid plaque to statin therapy, as progression of carotid atherosclerosis is associated with a paradoxical increase and regression of atherosclerosis with a paradoxical decrease in luminal area.

## Funding

Internal.

